# Risk factors associated with positive bacterial culture in salvaged red blood cells during cardiac surgery and postoperative infection incidence: A prospective cohort study

**DOI:** 10.3389/fmed.2023.1099351

**Published:** 2023-02-21

**Authors:** Yenong Zhou, Tao Chen, Chen Yang, Jincheng Liu, Xiuling Yang, Bing Zhang, Zhenxiao Jin

**Affiliations:** Department of Cardiovascular Surgery, Xijing Hospital, Fourth Military Medical University, Xi’an, China

**Keywords:** cardiac surgery, patient blood management (PBM), postoperative infection, intensive care, Cell Saver

## Abstract

**Background:**

This study was designed to explore factors associated with the incidence of *positive bacterial* culture of salvaged red blood cells (sRBCs) recovered with a Cell Saver instrument during cardiac surgery and the impact of such positive outcomes on postoperative infection-related morbidity.

**Methods:**

The cohort study enrolled 204 patients scheduled for cardiac surgery with intraoperative blood cell salvage and retransfusion from July 2021 to July 2022. These patients were stratified into two groups based on intraoperative sRBCs bacterial culture results: culture (+) and culture (−) groups. Preoperative and intraoperative variables were compared between these groups aim to detect possible predictors of positive culture in sRBCs. In addition, differences in postoperative infection-related morbidity and other clinical outcomes were compared between these groups.

**Results:**

Of these patients, 49% were sRBCs culture (+), with *Staphylococcus epidermidis* as the most commonly identified pathogen. Risk factors independently associated with the risk of positive culture in sRBCs included BMI ≥25 kg/m^2^, a history of smoking, an operative duration ≥277.5 min, the higher number of staff in the operating room and higher surgical case order. Patients in the sRBCs culture (+) group exhibited a longer average ICU stay [3.5 days (2.0–6.0) vs. 2 days (1.0–4.0), *P* < 0.01], a longer duration of ventilation [20.45 h (12.0–17.8) vs. 13 h (11.0–17.0, *P* = 0.02)], underwent more allogeneic blood transfusions, exhibited higher transfusion-related costs [2,962 (1,683.0–5,608.8) vs. 2,525 (1,532.3–3,595.0), *P* = 0.01], and had higher rates of postoperative infections (22 vs. 9.6%, *P* = 0.02) as compared to patients in the sRBCs culture (−) group. In addition, culture (+) in sRBCs was an independent risk factor for postoperative infection (OR 2.62, 95% CI 1.16–5.90, *P* = 0.02).

**Conclusion:**

*Staphylococcus epidermidis* was the most common pathogen detected in sRBCs in the culture (+) group in this study, identifying it as a potential driver of postoperative infection. Positive sRBCs culture may contribute to postoperative infection and its incidence was significantly associated with patient BMI, history of smoking, operative duration, the number of staff in the operating room and surgical case order.

## 1. Introduction

The utilization of large volumes of blood products is more likely to occur when patients undergo open heart surgery owing to the inherent complexity of cardiopulmonary bypass (CPB) and associated surgical procedures. Indeed, the 15–20% of patients undergoing cardiac procedures are estimated to consume over 80% of all blood products in operative settings (1). Patient blood management (PBM) is a multimodal, multidisciplinary approach that centers around three main goals: improving preoperative red blood cell (RBC) counts as through the early detection and treatment of anemia, minimizing intraoperative blood loss through strategies such as intraoperative cell salvage (ICS), and optimizing the patient-specific tolerance of anemia (2). PBM can alleviate the pressure placed on blood resources while also reducing rates of transfusion-related complications and contributing to improved postoperative patient outcomes (3). ICS and the infusion of sRBCs form key components of PBM strategies in cardiac surgery patients. Relative to allogeneic blood, sRBCs exhibit notable advantages including good oxygen-carrying capacity and a high anti-acid buffering rate, and it is more convenient and efficient than allogeneic blood when treating patients with rare blood types or individuals suffering from emergency hemorrhage (4).

Intraoperative cell salvage has become an essential life-saving technology when performing complex operations. However, the benefits of a given clinical strategy must be carefully weighed against the potential adverse outcomes of such treatment. Manuel detected Gram-positive bacteria, primarily *Staphylococcus epidermidis*, in sRBCs samples collected from shed blood following washing with Cell Saver (CS) machines in patients undergoing cardiac surgery (5). They further observed higher rates of postoperative infections in patients that underwent cardiac surgery with ICS as compared to cardiac surgery patients for whom salvage was not performed (6). Even so, a meta-analysis of over 4,000 patients found ICS to be associated with a 28.7% reduction in the overall risk of infective morbidity (7). As such, whether the transfusion of sRBCs with positive bacterial culture results is likely to impact patients remains a matter of some controversy (8). Prior studies of ICS use in patients undergoing cardiac surgery have largely failed to conduct an adequate evaluation of the associated costs and benefits of such treatment. Accordingly, this study was developed to assess rates of sRBCs culture positivity in cardiac surgery patients, risk factors associated with such positivity, and the relationship between sRBCs culture positivity and postoperative infectious morbidity in these patients.

## 2. Materials and methods

### 2.1. Study population

The Ethics Committee of Hospital approved the prospective cohort study, which was registered in the Chinese Clinical Trial Registry (ChiCTR2100050367). In total, 204 patients scheduled to undergo cardiac surgery with the use of a CS device between July 2021 and July 2022 were enrolled in this study. All patients who were over 18 years of age and provided informed consent were enrolled in the study. Patients were excluded if: (1) CS use was not planned based on the scheduled surgical indications, (2) patients exhibited contraindications for CS use such as immunosuppression, active infections, or cancer, (3) patients were undergoing antimicrobial drug treatment, as this had the potential to bias study results, or (4) patients were treated with antibiotics for more than 24 h before the operation. Patients were separated into two groups based on the intraoperative sRBCs bacterial culture results, including a culture (+) group and a culture (−) group.

### 2.2. Autologous blood transfusion

Cell Saver use was performed in strict accordance with standard procedures. In this experiment, a Cell Saver Elite (Haemonetics, USA) was used in the operation. Heparinized saline solution with 25.000 IU of heparin in 1 L of 0.9% saline solution at a rate of 100 ml/h was used to prevent thrombogenesis during blood collection. During non-heparinized periods, any blood shed from the wound and mediastina was heparinized and drawn into the reservoir of the CS device *via* negative pressure (<150 mmHg). Salvaged blood was then filtered, centrifuged, washed, and concentrated to sRBCs that were transfused back into the patient as appropriate (9). For patients who need CPB, the target flow rate was 2.4 L/(m^2^/min), and CPB was initiated when the activated clotting time (ACT) was greater than 480 s. When the patient’s temperature had reached 36°C, they were gradually weaned off of CPB and protamine was used at a 1:1 ratio to neutralize heparin. After CPB, the residual blood in the pipeline and CPB is also washed by CS and returned to the patient. Intravenous rocuronium, sufentanil, propofol, and midazolam were used for anesthesia induction, whereas maintenance anesthesia throughout the procedure consisted of sufentanil, pipecuronium, and midazolam. RBCs transfusion during CPB is jointly decided by the surgeon, anesthetist, and perfusionist according to the patient’s condition. Transfusion of RBCs when postoperative hemoglobin level is below 80 g/L. Transfusion of fresh frozen plasma, platelets, and cold precipitation when bleeding is excessive.

### 2.3. Patient grouping and blood sample collection

Data in this study were compiled prospectively, and all blood samples were collected in accordance with appropriate aseptic techniques. At 30 min before surgery, venous blood samples were collected from patients for bacterial culture. Culture results for intraoperatively collected sRBCs at the time after washing and concentration, but before transfusion were used to group patients into a culture (+) group and a culture (−) group. Venous blood was also collected from all patients on postoperative day 1 and 3 for bacterial culture. All blood samples were added to BD BACTEC adult aerobic and anaerobic culture bottles (Plus Aerobic/Anaerobic, BD, USA), which were then gently inverted and mixed to prevent clotting, after which they were subject to timely examination. A total of 8–10 ml of blood was collected for each bottle, and these bottles were then incubated in the automated BD BACTE-CFX400 blood culture instrument (BD, USA). When a positive alarm was observed, samples of culture fluid were collected for Gram staining and culture on blood agar plates at 35°C for 24–48 h in a CO_2_ incubator. A fully automated VITEK MS mass spectrometer (bioMérieux, France) was used for bacterial species identification.

### 2.4. Definitions

Postoperative infection data were prospectively collected and classified based on the Centers for Disease Control and the National Healthcare Safety Network (CDC/NHSN) criteria (10). When patients exhibited more than one infection, the earliest detected infection was included in the present analyses. This study primarily focused on major infections including cases of (1) pneumonia; (2) surgical site infection (SSI); (3) urinary tract infection (UTI); and (4) bloodstream infection. Pneumonia was diagnosed based on positive X-ray results together with at least one of the following: (1) fever >38°C without any other cause, (2) altered mental status in individuals ≥70 years, and (3) a leukocyte count <4,000 or >12.000 WBC/mm^3^, together with one or more of the following: (1) new-onset purulent sputum production or changes in sputum characteristics, (2) new-onset or worsening bronchial breath sounds, (3) worsening gas exchange (O_2_ desaturation, increased O_2_ requirements, or increased ventilation demand), and (4) new-onset or worsening cough, dyspnea, or tachypnea. SSI cases were defined by infections occurring within 30 days after surgery that met at least one of the following criteria, while also applied for cases of mediastinitis and organ/space SSI: (1) purulent drainage from the surgical incision, (2) the isolation of microorganisms from the surgical incision, (3) symptoms of infection, or (4) diagnosis by a surgeon or attending physician. UTIs were diagnosed based on the following symptoms: (1) fever >38°C, (2) positive urine microbial culture results with positive clinical signs, and (3) a physician diagnosis of a UTI or the administration of treatment appropriate for a UTI. Bloodstream infections were diagnosed in patients meeting at least one of the following criteria: positive blood culture results, fever (>38°C), chills, or hypotension with positive laboratory results.

### 2.5. Statistical analysis

Similar prior studies have reported a 70% frequency of positive bacterial culture in sRBCs (11). Accordingly, sample size calculations for this study were designed to detect a 70% frequency with a 5% margin of error and an 85% confidence interval, leading to the determination that 173 patients would be required for this study. Assuming a dropout rate of 10%, the target patient population was 193. All power calculations were performed using PASS 25 (IBM, NY, USA). The Kolmogorov–Smirnov test was used to determine whether data were normally distributed. Categorical variables were reported as frequencies (%) and compared *via* Chi-square tests and Fisher’s exact test. Normally distributed continuous variables are reported as means [±standard deviation (SD)] and were compared with unpaired *t*-tests, whereas non-normally distributed variables are given as the median [interquartile range (IQR)] and compared with the Mann–Whitney U test. Univariate logistic regression analyses were initially used to screen for risk factors, with those factors exhibiting a *P* < 0.1 in univariate analyses being incorporated into a forward stepwise multivariate logistic regression analysis exploring independent predictors. Data are reported as odds ratios (ORs) and 95% confidence intervals (CIs). The cut-off value for operative duration and CS usage time were selected using a receiving operating curve based on the value that achieved the maximal sum of sensitivity and specificity (277.5 and 272.5 min in this study population). BMI classification was performed based on standard guidelines (5). SPSS 26.0 (IBM, NY, USA) was used for all statistical analyses, with *P* < 0.05 as the significance threshold.

## 3. Results

### 3.1. Study population

**FIGURE 1:**
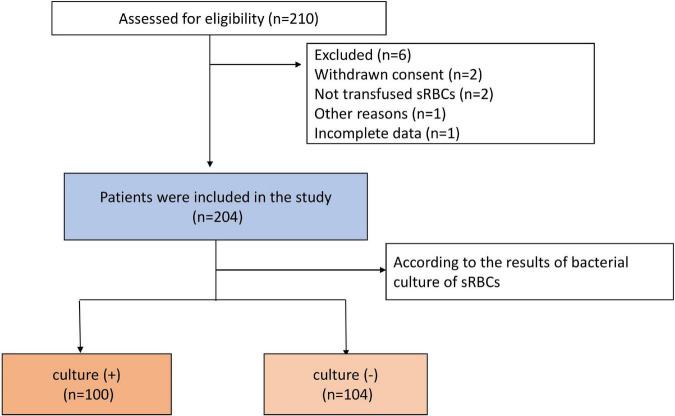
Study flowchart.

In total, 210 patients underwent cardiac surgery with ICS use over the study period (July 2021 – July 2022). Four patients were excluded due to their sRBCs having not been transfused or the withdrawal of consent, while two patients were excluded due to incomplete data or other reasons. The remaining 204 patients were included in this study, of whom 100 were included in the culture (+) group and 104 were included in the culture (−) group. A flowchart for this study is provided in Figure 1. There were statistically significant differences in BMI and smoking history between the groups. Patients in the culture (+) group exhibited a longer average operative duration (*P* < 0.01) and were more likely to exhibit a No. 2 surgical case order (*P* < 0.01) as compared to patients in the culture (−) group. Patients that underwent surgical procedures necessitating CPB were more likely to be in the culture (+) group (*P* = 0.02), and these patients exhibited a longer duration of CPB (*P* < 0.01), longer duration of aortic cross-clamping (*P* < 0.01), and longer duration of the CS usage (*P* < 0.01) relative to culture (−) patients. Patients in the culture (+) group required more intraoperative allogeneic RBCs (*P* < 0.01) and plasma (*P* < *0.01*). The maximum number of people in the operating room was also higher in the culture (+) group relative to the culture (−) group (*P* = 0.01). For details regarding patient characteristics, see Table 1.

**TABLE 1:** Patient baseline and perioperative characteristics.

Variable	Culture (+) (*n* = 100)	Culture (−) (*n* = 104)	*P*-value
**Demographics**			
Male sex	74 (74.0)	76 (73.1)	0.88
Age (year)	56 (46.0–62.8)	54 (43.0–60.8)	0.47
BMI			<0.01
<25	45 (45.0)	67 (64.4)	
≥25	55 (55.0)	37 (35.6)	
Smoking history	53 (53.0)	35 (33.7)	<0.01
Drinker	37 (37.0)	37 (35.6)	0.83
**Comorbidities**			
Diabetes	24 (24.0)	22 (21.2)	0.63
Hypertension	34 (34.0)	35 (33.7)	0.96
Pulmonary disease	7 (7.0)	8 (7.7)	0.85
Previous cerebrovascular event	5 (5.0)	6 (5.8)	0.81
History of cardiac surgery	7 (7.0)	3 (2.9)	0.17
Kidney disease	3 (3.0)	5 (4.8)	0.72
Liver disease	3 (3.0)	4 (3.8)	1.00
**EuroSCORE II (%)**	2 (1.4–3.1)	2 (1.1–3.4)	0.10
**Type of surgery**			
Valve only	41 (41.0)	45 (43.3)	0.74
CABG only	16 (16.0)	22 (21.2)	0.35
CABG and valve	6 (6.0)	11 (10.6)	0.24
Major aortic procedure	37 (37.0)	26 (25.0)	0.06
**Duration of surgery (min)**	338 (280.0–392.5)	245 (200.0–327.5)	<0.01
**Surgical case order**			<0.01
No. 1	59 (59.0)	80 (76.9)	
No. 2	41 (41.0)	24 (23.1)	
**CPB**			0.02
On CPB	88 (88.0)	78 (75.0)	
Off CPB	12 (12.0)	26 (25.0)	
Duration of CPB (min)	178 (124.0–226.8)	121 (77.8–160.5)	<0.01
Duration of aortic cross-clamping (min)	86 (68.3–115.8)	61 (30.0–83.8)	<0.01
**Allogeneic blood transfusion during operation**			
RBC (U)	1.75 (0–4.0)	0 (0–1.5)	<0.01
Fresh frozen plasma (ml)	300 (0–600.0)	0 (0–397.5)	<0.01
Cold precipitation (U)	0 (0–9.0)	0 (0–0)	0.35
Platelet (U)	0 (0–0)	0 (0–0)	0.20
**Cell Saver**			
Vol. processed (ml)	875 (669.3–1,233.8)	750 (525.0–1,278.5)	0.12
Vol. salvaged (ml)	312 (217.3–430.0)	240 (210.0–425.5)	0.30
Duration of the usage (min)	292.5 (210.0–357.7)	**202.5 (180.0–257.5)**	**<0.01**
**Number of people in the operating room (max)**	**9 (6.3–10.0)**	**7 (6.0–9.0)**	**0.01**
**Appropriate timing of preoperative antibiotics**	**95 (95.0)**	**100 (96.2)**	**0.75**
**Intraoperative antibiotic redosed after 3 h**	**98 (98.0)**	**103 (99.0)**	**0.62**
**Postoperative antibiotic (co-medication)**	**25 (25.0)**	**20 (19.2)**	**0.32**

Non-normally distributed continuous variables are reported as the median (IQR). Categorical variables are reported as *n* (%). BMI, body mass index; CABG, coronary artery bypass graft; CPB, cardiopulmonary bypass; RBC, red blood cell; Vol., volume; No., number.

### 3.2. Risk factors associated with culture (+) in sRBCs

Factors exhibiting a *P* < 0.1 in univariate analyses were incorporated into a forward stepwise multivariate logistic regression analysis (Table 2). Factors found to be independently associated with positive sRBCs culture included BMI ≥25, smoking history, duration of surgery ≥277.5 min, more staff in the operating room, and higher surgical case order.

**TABLE 2:** Univariable analyses and multivariable models for independent factors associated with positive bacterial culture in sRBCs.

	Univariate analysis			Multivariable analysis		
	***P*-value**	**OR**	**95% CI**	***P*-value**	**OR**	**95% CI**
Male sex	0.88	1.05	0.56–1.95			
Age (year)	0.63	1.00	0.98–1.03			
BMI ≥25	0.01	2.21	1.26–3.88	0.03	2.08	1.08–4.00
Smoking history	0.01	2.22	1.26–3.91	0.04	1.99	1.03–3.86
Drinker	0.83	1.06	0.60–1.88			
Diabetes	0.63	1.18	0.61–2.27			
Hypertension	0.96	1.02	0.57–1.81			
Pulmonary disease	0.85	0.90	0.32–2.59			
Previous cerebrovascular event	0.94	1.04	0.33–3.35			
History of cardiac surgery	0.19	2.53	0.64–10.09			
Kidney disease	0.51	0.61	0.14–2.63			
Liver disease	0.74	0.77	0.17–3.55			
EuroSCORE II (%)	0.74	0.77	0.17–3.55			
Major aortic procedure	0.07	1.76	0.97–3.22			
Duration of surgery ≥277.5 (min)	<0.01	5.81	3.64–12.75	<0.01	5.58	2.86–10.86
Higher surgical case order	<0.01	2.41	1.32–4.42	0.04	2.07	1.03–4.17
On CPB	0.02	2.44	1.16–5.17			
Duration of CPB (min)	<0.01	1.01	1.00–1.01			
Duration of aortic cross-clamping (min)	<0.01	1.01	1.01–1.02			
Number of people in the operating room (max)	<0.01	1.18	1.06–1.32	0.01	1.19	1.05–1.36
**Allogeneic blood transfusion during operation**						
RBC (U)	<0.01	1.01	1.01–1.02			
Fresh frozen plasma (ml)	<0.01	1.01	1.00–1.00			
Cold precipitation (U)	0.38	1.03	0.96–1.10			
Platelet (U)	0.28	0.55	0.18–1.62			
**Cell Saver**						
Vol. processed (ml)	0.54	1.00	0.99–1.00			
Vol. salvaged (ml)	0.94	1.00	0.99–1.00			
Duration of the usage ≥272.5 (min)	<0.01	5.15	2.78–9.53			

BMI, body mass index; RBC, red blood cell; OR, odds ratio; CI, confidence interval.

### 3.3. Identification of microbes present in sRBCs

Bacteria were identified in 49% of sRBCs samples. Gram-positive bacteria were the most frequently isolated, with *S. epidermidis* species being the most common and present in 64% (*n* = 64) of cases (Table 3).

**TABLE 3:** Bacterial species identified cases of sRBCs culture positivity.

Culture (+) group	Fr (%)*
**Gram-positive**	
*S. epidermidis*	64 (64.0)
*S. haemolyticus*	6 (6.0)
*S. caprae*	3 (3.0)
*S. hominis*	8 (8.0)
*S. cohnii*	2 (2.0)
*S. capitis*	5 (5.0)
*S. saccharolyticus*	4 (4.0)
*M. luteus*	3 (3.0)
*Str. viridans*	2 (2.0)
*C. diphtheriae*	1 (1.0)
**Gram-negative**	
*B. fragilis*	1 (1.0)
*A. ursingii*	1 (1.0)

*Fr (%), frequency (percentages). *S., Staphylococcus; Str., Streptococci; C., Corynebacterium; B., Bacteroides; M., Micrococcus; A., Acinetobacter.*

### 3.4. Postoperative patient outcomes

Patients in the culture (+) group exhibited a significantly longer ventilation time (*P* = 0.02) and a longer duration of ICU stay (*P* < 0.01) relative to the culture (−) group. Patients in the culture (+) group also required more allogeneic blood and had higher transfusion-related costs than patients in the culture (−) group. With respect to postoperative infection incidence, only the rate of bloodstream infections differed significantly between these groups (*P* = 0.03). For further details see Table 4.

**TABLE 4:** Postoperative patient outcome data.

	Culture (+) (*n* = 100)	Culture (−) (*n* = 104)	*P*-value
ICU length of stay (day)	3.5 (2.0–6.0)	2 (1.0–4.0)	<0.01
Hospital length of stay (day)	14 (12.0–17.8)	13 (11.0–17.0)	0.27
Ventilator duration (h)	20.45 (15.5–36.6)	20.0 (10.1–26.0)	0.02
Re-exploration	4 (4.0)	6 (5.8)	0.56
30-day mortality	2 (2.0)	4 (3.8)	0.68
Total cost × 10^5^ (¥)	2 (1.2–1.9)	1.3 (1.1–1.9)	0.07
Blood product cost (¥)	2,962 (1,683.0–5,608.8)	2,525 (1,532.3–3,595.0)	0.01
**Allogeneic blood transfusions after surgery**			
RBC (U)	2 (0–6.4)	1.75 (0–4.0)	0.03
Fresh frozen plasma (ml)	1,355 (800.0–2,295.0)	1,175 (700.0–1,595.0)	0.03
Cold precipitation (U)	0 (0–8.0)	0 (0–0)	0.10
Platelet (U)	0 (0–0)	0(0–0)	0.79
**Positive blood culture**			
Preoperative	2 (2.0)	1 (1.0)	0.62
The first postoperative day	2 (2.0)	1 (1.0)	0.62
The third postoperative day	10 (10.0)	3 (2.9)	0.04
**Postoperative infections**	22 (22.0)	10 (9.6)	0.02
Pneumonia	6 (6.0)	2 (1.9)	0.16
Bloodstream infection	12 (12.0)	4 (3.8)	0.03
Surgical site infection	3 (3.0)	2 (1.9)	0.68
Urinary tract infection	1 (1.0)	2 (1.9)	1.00

Non-normally distributed continuous variables are reported as the median (IQR). Categorical variables are reported as *n* (%).

### 3.5. Risk factors associated with postoperative infection

At last, we wondered whether culture (+) in sRBCs was an independent risk factor for postoperative infection. Univariable analyses and multivariable models for independent factors associated with postoperative infection were processed. According to univariable analyses, advanced age (*P* = 0.03), BMI ≥25 (*P* = 0.08), pulmonary disease (*P* = 0.06), combined valve and CABG procedures (*P* = 0.09), more allogeneic RBC transfusions during surgery (*P* = 0.09), sRBCs positive (*P* = 0.02), and longer ICU length of stay (*P* = 0.01) were significantly associated with postoperative infection. Among these above factors, only eldly and sRBCs positive independently contributed to the development of postoperative infection as shown in forward multivariate logistic regression analysis (Table 5).

**TABLE 5:** Univariable analyses and multivariable models for independent factors associated with postoperative infection.

	Univariate analysis			Multivariable analysis		
	*P*-value	OR	95% CI	*P*-value	OR	95% CI
Male sex	0.84	1.10	0.46–2.61			
Age (year)	0.03	1.04	1.00–1.08	0.04	1.04	1.00–1.08
BMI ≥25	0.08	1.98	0.92–4.27			
Smoking history	0.40	1.39	0.65–2.96			
Drinker	0.34	1.45	0.68–3.12			
Diabetes	0.20	1.72	0.75–3.95			
Hypertension	0.46	0.73	0.32–1.68			
Pulmonary disease	0.06	3.00	0.95–9.46			
Previous cerebrovascular event	0.92	1.08	0.23–5.18			
History of cardiac surgery	0.70	1.37	0.28–6.75			
Kidney disease	0.47	1.84	0.36–9.57			
Liver disease	0.92	0.89	0.10–7.67			
EuroSCORE II (%)	0.36	1.13	0.87–1.46			
Duration of surgery	0.58	1.00	1.00–1.01			
Higher surgical case order	0.62	0.81	0.35–1.87			
On CPB	0.99	0.99	0.38–2.61			
Culture (+) in sRBCs	0.02	2.65	1.19–5.93	0.02	2.62	1.16–5.90
ICU length of stay (day)	0.01	1.14	1.03–1.25			
Hospital length of stay (day)	0.39	0.97	0.90–1.04			
Ventilator duration (h)	0.94	1.00	0.99–1.01			
Re-exploration	0.62	0.58	0.07–4.78			
**Type of surgery**						
Valve only	Reference group					
CABG only	0.66	1.28	0.44–3.76			
Valve and CABG	0.09	2.84	0.84–9.62			
Major aortic procedure	0.59	1.29	0.51–3.25			
**Allogeneic blood transfusion during operation**						
RBC (U)	0.08	0.82	0.65–1.03			
Fresh frozen plasma (ml)	0.59	1.00	1.00–1.01			
**Allogeneic blood transfusions after surgery**						
RBC (U)	0.41	1.02	0.97–1.07			
Fresh frozen plasma (ml)	0.41	1.00	1.00–1.00			

BMI, body mass index; CABG, coronary artery bypass graft; CPB, cardiopulmonary bypass; RBC, red blood cell; Vol., volume; OR, odds ratio; CI, confidence interval.

## 4. Discussion

This study was developed to explore the incidence of positive bacterial culture in sRBCs in the context of cardiac surgery, to identify predictors of positivity, and to evaluate its impact on postoperative infection incidence in these patients in an effort to guide the better prevention of microbial.

In the 2017 EACS/EACTA guidelines, CS has a category IIa recommendation for routine use as a component of PBM strategies during cardiac surgery in adult patients with the goal of minimizing the need for allogeneic blood products (12). While advantageous, however, this savage strategy has been shown to reduce coagulation factors and contribute to higher rates of postoperative infective morbidity (13, 14). This is particularly important given that infections are the most common non-cardiac complication following cardiac surgical procedures and they have a pronounced impact on patient survival and readmission rates (15). Accordingly, this study was designed to explore the relationship between CS and infection.

The washing process employed by CS devices has been shown to remove the majority of bacteria from sRBCs preparations (16–18), but 10–30% of the bacteria may remain after washing (8). Here, positive bacterial cultures were obtained for 49% of cardiac surgery patient sRBCs samples, with *S. epidermidis* being the most prevalent microbe in these samples. *S. epidermidis* is generally a harmless commensal bacterium that is commonly detected on the human skin and mucosal surfaces, and that can also readily adhere to medical devices such that it represents a clinically important opportunistic pathogen. As it can readily form a biofilm, *S. epidermidis* can resist antibiotic treatment, contributing to persistent or prolonged infections. The majority of bacteria isolated from bloodstream infection patients are Gram-positive bacteria, including coagulase (−) bacteria such as *S. epidermidis* (19), which is a common causative agent in cases of nosocomial blood infections (20). *S. epidermidis* was previously reported to be associated with 69% of culture (+) in sRBCs following the CS-mediated washing of salvaged blood for patients undergoing cardiac surgery. But it did not demonstrate the effect on patients after surgery (5). In contrast, the present results offer insight into postoperative infection incidence in patients that were transfused with bacterially positive sRBCs.

It remains controversial as to whether the use of CS devices resulting in positive sRBCs culture contributes to higher infective morbidity. One report demonstrated that RBCs transfusion was associated with infections and that relative to a liberal transfusion strategy, a more restrictive strategy was associated with a lower risk of infection (21). In a study of cardiac surgery patients, ICS was directly associated with increased infective morbidity, although the surgery types in that study were restricted to CABG and valvular surgery (6). A separate report observed no adverse patient outcomes despite the frequent contamination of sRBCs with Gram-positive skin-derived bacteria commensal species and low levels of endotoxin, although that study is relatively old and had a small sample size (22). In the present study, significant differences in the length of ICU stay, duration of ventilation, and postoperative bloodstream infection rates were observed between patients with and without detectable positive bacterial culture in sRBCs. However, our study found that the bacteria cultured in sRBCs were not completely consistent with the bacteria cultured in postoperative infected patients, which seems to weaken the link between sRBCs culture positive and postoperative infection. However, forward univariable and multivariable logistic regression analyses for independent factors associated with postoperative infection also indicated that culture (+) in sRBCs is the independent risk factor for contributing to postoperative infection in this study. These findings thus suggest that positive sRBCs bacteria culture results are indicative of at least some level of safety risk.

Previous reports have explored risk factors associated with culture (+) in sRBCs, yielding varying outcomes that may be attributable to differences in patients, exposure time, intraoperative procedures, and the surgical environment (including operating room cleanliness). One study of cardiac surgery patients found that those with a BMI ≥25 who underwent valvular surgery tended to be more susceptible to *S. epidermidis* cultures (+) in sRBCs (5). Another controlled study compared new and old operating rooms and detected fivefold lower bacterial counts in sRBCs samples prepared in the new operating room relative to the old room, indicating that operating room cleanliness is likely to impact bacterial deposition (23). Both longer operative duration and higher case order have previously been established as significant infection-related risk factors (24, 25). Those patients with a BMI ≥25 and a preoperative history of smoking were more likely to exhibit culture (+) in sRBCs. In these patients, preadmission education can be tailored toward the promotion of dietary control, smoking cessation, and appropriate cleaning procedures, while postoperative efforts should focus on monitoring for a range of infection-related indicators so that vigorous treatment can be rapidly deployed when appropriate. The present results also support the status of longer operative duration, higher surgical case order, and more staff in the operating room as independent predictors of positive bacterial culture in sRBCs. Contaminant accumulation during an initial case has been shown to be perpetuated and amplified for subsequent cases, as in one example in which 0 and 7% of the operating room was found to be contaminated prior to the treatment of patient 1 and patient 2, respectively (26). This is consistent with the present results demonstrating that higher case order and higher levels of contamination may contribute to bacterial transmission. Higher volumes of operating room traffic due to staffing changes can further increase this risk, and the reduction of unnecessary traffic by assigning staff to particular cases rather than shifts could potentially mitigate such contamination and associated microbial transmission (24). Most importantly, it is vital that standardized operating room nursing procedures be developed and that operating room management be conducted carefully. This includes outpatient and pre-admission education, the strict and timely administration of antibiotics, the monitoring of operating room air quality, the sterilization of surgical instruments, the minimization of the operative duration, and the elimination of operating room traffic wherever possible. Implementing comprehensive nursing interventions for risk factors can effectively enhance patient prognostic outcomes and quality of life.

This study is subject to some limitations, including the fact that the study population was relatively small. Recent reports have suggested that adding vancomycin can eliminate the bacterial of sRBCs when using CS devices (27), but this approach remains to be tested in the context of cardiac surgery. In future studies, we will focus on actively collaborating with other centers to increase the study sample size and to conduct bacterial sensitivity testing aimed at identifying the lowest concentrations of the most effective antibiotics capable of lowering rates of culture positivity.

## 5. Conclusion

The present results confirmed that positive bacterial culture in sRBCs is evident in cardiac surgery patients, and that was associated with patient BMI, history of smoking, operative duration, operating room traffic, and surgical case order. The use of these positive sRBCs was, in turn, associated with postoperative infections and other adverse outcomes.

## Data availability statement

The raw data supporting the conclusions of this article will be made available by the authors, without undue reservation.

## Ethics statement

This trial was reviewed and approved by the Ethics Committee of Xijing Hospital, which has been registered in the Chinese Clinical Trial Registry (ChiCTR2200060439). All the patients signed an informed consent form. The patients/participants provided their written informed consent to participate in this study.

## Author contributions

JL, BZ, and ZJ designed the clinical study. YZ, XY, and TC collected the clinical data. YZ, CY, and TC analyzed the data and drafted this manuscript. YZ, BZ, and ZJ modified the manuscript. All authors revised and approved the final manuscript.

## References

[1] Ferraris V Ferraris S Saha S Hessel E Haan C Royston B Perioperative blood transfusion and blood conservation in cardiac surgery: the society of thoracic surgeons and the society of cardiovascular anesthesiologists clinical practice guideline. *Ann Thoracic Surg.* (2007) 83:S27–86. 10.1016/j.athoracsur.2007.02.099 17462454

[2] Leahy M Hofmann A Towler S Trentino K Burrowa S Swain S Improved outcomes and reduced costs associated with a health-system-wide patient blood management program: a retrospective observational study in four major adult tertiary-care hospitals. *Transfusion.* (2017) 57:1347–58. 10.1111/trf.14006 28150313

[3] Nwafor I Arua O Eze J Ezemba N Nwafor M. Utilisation of blood and blood products during open heart surgery in a low-income country: our local experience in 3 years. *Cardiol Young.* (2018) 28:1289–94. 10.1017/S1047951118001269 30070188

[4] Frank S Sikorski R Konig G Tsilimigras D Hartmann J Popovsky M Clinical utility of autologous salvaged blood: a review. *J Gastrointest Surg.* (2020) 24:464–72. 10.1007/s11605-019-04374-y 31468332

[5] Manuel L. Bacteremia in the red blood cells obtained from the cell saver in patients submitted to heart surgery. *Rev Latino-Am Enfermagem.* (2020) 28:e3337. 10.1590/1518-8345.3092.3337 32876294 PMC7458575

[6] Klarenbosch J Heuvel E Oeveren W Vries A. Does intraoperative cell salvage reduce postoperative infection rates in cardiac surgery? *J Cardiothor Vasc Anesth.* (2020) 34:1457–63. 10.1053/j.jvca.2020.01.023 32144053

[7] Klein A Bailey C Charlton A Evans E Guckian-Fisher M McCrossan R. Association of anaesthetists guidelines: cell salvage for peri-operative blood conservation 2018. *Anaesthesia.* (2018) 73:1141–50. 10.1111/anae.14331 29989144

[8] Bolliger D Fassl J. Less transfusion, less infections-controversies in patient blood management. *J Cardiothor Vasc Anesth.* (2020) 34:1464–6. 10.1053/j.jvca.2020.02.037 32241679

[9] Vonk A Meesters M Garnier R Romijin J van Barneveld L Heymans M Intraoperative cell salvage is associated with reduced postoperative blood loss and transfusion requirements in cardiac surgery: a cohort study. *Transfusion.* (2013) 53:2782–9. 10.1111/trf.12126 23445352

[10] Horan T Andrus M Dudeck MA. CDC/NHSN surveillance definition of health care-associated infection and criteria for specific types of infections in the acute care setting. *Am J Infect Control.* (2008) 36:309–32. 10.1016/j.ajic.2008.03.002 18538699

[11] Shindo S Matsumoto H Kojima A Kubota K Matsumoto M. Temporary bacteremia due to intraoperative blood salvage during cardiovascular surgery. *Am J Surg.* (2004) 188:237–9. 10.1016/j.amjsurg.2004.03.008 15450826

[12] Boer C Meesters M Milan M Benedetto U Bolliger D von Heymann C 2017 EACTS/EACTA guidelines on patient blood management for adult cardiac surgery. *J Cardiothorac Vasc Anesth.* (2018) 32:88–120. 10.1053/j.jvca.2017.06.026 29029990

[13] Burman J Westlake A Davidson S Rutherford L Rayner A Wright A Study of five cell salvage machines in coronary artery surgery. *Transfus Med.* (2002) 12:173–9. 10.1046/j.1365-3148.2002.00369.x 12071873

[14] Kiser K Tanaka A Sandhu H Miller C Leonard S Safi H Extensive cell salvage and postoperative outcomes following thoracoabdominal and descending aortic repair. *J Thorac Cardiovasc Surg.* (2022) 163:914–21. 10.1016/j.jtcvs.2020.06.005 32711982

[15] Gelijns A Moskowitz A Acker M Argenziano M Geller N Puskas J Management practices and major infections after cardiac surgery. *J Am Coll Cardiol.* (2014) 64:372–81. 10.1016/j.jacc.2014.04.052 25060372 PMC4222509

[16] Waters J Tuohy M Hobson D Procop G. Bacterial reduction by cell salvage washing and leukocyte depletion filtration. *Anesthesiology.* (2003) 99:652–5. 12960550 10.1097/00000542-200309000-00021

[17] Hinson W Rogovskyy A Lawhon S Mankin K. Influence of a cell salvage washing system and leukocyte reduction filtration on bacterial contamination of canine whole blood ex vivo. *Vet Surg.* (2020) 49:989–96. 10.1111/vsu.13410 32166777

[18] Waters J. Cell salvage in trauma. *Curr Opin Anaesthesiol.* (2021) 34:503–6. 10.1097/ACO.0000000000001014 34074882

[19] Matthaios P Kolonitsiou F Karamouzos K Tsilipounidaki K Nikolopoulou A Fligou F Molecular characteristics and predictors of mortality among gram-positive bacteria isolated from bloodstream infections in critically ill patients during a 5-year period (2012-2016). *Eur J Clin Microbiol Infect Dis.* (2020) 39:863–9. 10.1007/s10096-019-03803-9 31898796 PMC7223776

[20] Dawit T Mengesha R Ebrahim M Tequare M Abraha H. Nosocomial sepsis and drug susceptibility pattern among patients admitted to adult intensive care unit of ayder comprehensive specialized hospital, northern ethiopia. *BMC Infect Dis.* (2021) 21:824. 10.1186/s12879-021-06527-4 34404343 PMC8369143

[21] Rohde J Dimcheff D Blumberg N Saint S Langa K Kuhn L Health care-associated infection after red blood cell transfusion: a systematic review and meta-analysis. *JAMA.* (2014) 311:1317–26. 10.1001/jama.2014.2726 24691607 PMC4289152

[22] Bland L Villarino M Arduino M McAllister S Gordon S Uyeda C Bacteriologic and endotoxin analysis of salvaged blood used in autologous transfusions during cardiac operations. *J Thorac Cardiovasc Surg.* (1992) 103:582–8. 1545559

[23] Ishida T Nakano K Nakatani H Gomi A. Bacteriological evaluation of the cardiac surgery environment accompanying hospital relocation. *Surg Today.* (2006) 36:504–7. 10.1007/s00595-006-3178-9 16715418

[24] Gruskay J Kepler C Smith J Radcliff K Vaccaro A. Is surgical case order associated with increased infection rate after spine surgery? *Spine.* (2012) 37:1170–4. 10.1097/BRS.0b013e3182407859 22089398

[25] Robich M Sabik J Houghtaling P Kelava M Gordo S Blackstone E Prolonged effect of postoperative infectious complications on survival after cardiac surgery. *Ann Thoracic Surg.* (2015) 99:1591–9. 10.1016/j.athoracsur.2014.12.037 25686669

[26] Loftus R Muffly M Brown J Beach M Koff M Corwin H Hand contamination of anesthesia providers is an important risk factor for intraoperative bacterial transmission. *Anesth Anal.* (2011) 112:98–105. 10.1213/ANE.0b013e3181e7ce18 20686007

[27] Perez-Ferrer A Gredilla-Díaz E de Vicente-Sánchez J Navarro-Suay R Gilsanz-Rodríguez F. Vancomycin added to the wash solution of the cell-saver. Effect on bacterial contamination. *Rev Esp Anestesiol Reanim.* (2017) 64:185–91. 10.1016/j.redar.2016.10.002 28094033

